# Comparative Genomics of Cluster O Mycobacteriophages

**DOI:** 10.1371/journal.pone.0118725

**Published:** 2015-03-05

**Authors:** Steven G. Cresawn, Welkin H. Pope, Deborah Jacobs-Sera, Charles A. Bowman, Daniel A. Russell, Rebekah M. Dedrick, Tamarah Adair, Kirk R. Anders, Sarah Ball, David Bollivar, Caroline Breitenberger, Sandra H. Burnett, Kristen Butela, Deanna Byrnes, Sarah Carzo, Kathleen A. Cornely, Trevor Cross, Richard L. Daniels, David Dunbar, Ann M. Findley, Chris R. Gissendanner, Urszula P. Golebiewska, Grant A. Hartzog, J. Robert Hatherill, Lee E. Hughes, Chernoh S. Jalloh, Carla De Los Santos, Kevin Ekanem, Sphindile L. Khambule, Rodney A. King, Christina King-Smith, Karen Klyczek, Greg P. Krukonis, Christian Laing, Jonathan S. Lapin, A. Javier Lopez, Sipho M. Mkhwanazi, Sally D. Molloy, Deborah Moran, Vanisha Munsamy, Eddie Pacey, Ruth Plymale, Marianne Poxleitner, Nathan Reyna, Joel F. Schildbach, Joseph Stukey, Sarah E. Taylor, Vassie C. Ware, Amanda L. Wellmann, Daniel Westholm, Donna Wodarski, Michelle Zajko, Thabiso S. Zikalala, Roger W. Hendrix, Graham F. Hatfull

**Affiliations:** 1 Department of Biology, James Madison University, Harrisonburg, Virginia, United States of America; 2 Department of Biological Sciences, University of Pittsburgh, Pittsburgh, Pennsylvania, United States of America; 3 Department of Biology, Baylor University, Waco, Texas, United States of America; 4 Department of Biology, Gonzaga University, Spokane, Washington, United States of America; 5 Center for Life Sciences Education, The Ohio State University, Columbus, Ohio, United States of America; 6 Biology Department, Illinois Wesleyan University, Bloomington, Illinois, United States of America; 7 Department of Microbiology & Molecular Biology, Brigham Young University, Provo, Utah, United States of America; 8 Biology Department, Seton Hill University, Greensburg, Pennsylvania, United States of America; 9 Biology Department, Carthage College, Kenosha, Wisconsin, United States of America; 10 Department of Chemistry & Biochemistry, Providence College, Providence, Rhode Island, United States of America; 11 Biology Department, College of Idaho, Caldwell, Idaho, United States of America; 12 Department of Biology, Cabrini College, Radnor, Pennsylvania, United States of America; 13 School of Sciences, University of Louisiana at Monroe, Monroe, Louisiana, United States of America; 14 School of Pharmacy, University of Louisiana at Monroe, Monroe, Louisiana, United States of America; 15 Department of Biological Sciences & Geology, Queensborough Community College, Bayside, New York, United States of America; 16 Department of Molecular, Cell & Developmental Biology, University of California Santa Cruz, Santa Cruz, California, United States of America; 17 Department of Natural Sciences, Del Mar College, Corpus Christi, Texas, United States of America; 18 Department of Biological Sciences, University of North Texas, Denton, Texas, United States of America; 19 School of Life Sciences, University of QwaZulu-Natal, Durban, South Africa; 20 Department of Biology, Western Kentucky University, Bowling Green, Kentucky, United States of America; 21 Department of Biology, Saint Joseph’s University, Philadelphia, Pennsylvania, United States of America; 22 Department of Biology, University of Wisconsin-River Falls, River Falls, Wisconsin, United States of America; 23 Department of Biology, Gettysburg College, Gettysburg, Pennsylvania, United States of America; 24 Department of Math & Computer Science, Wilkes University, Wilkes Barre, Pennsylvania, United States of America; 25 Department of Biological Sciences, Carnegie Mellon University, Pittsburgh, Pennsylvania, United States of America; 26 Department of Molecular & Biomedical Sciences, University of Maine Honors College, Orono, Maine, United States of America; 27 KwaZulu-Natal Research Institute for Tuberculosis & HIV, Durban, South Africa; 28 Department of Biological Sciences, Ouachita Baptist University, Arkadelphia, Arkansas, United States of America; 29 Department of Biology, Johns Hopkins University, Baltimore, Maryland, United States of America; 30 Department of Biology, Hope College, Holland, Michigan, United States of America; 31 Department of Biology, Brown University, Providence, Rhode Island, United States of America; 32 Department of Biological Sciences, Lehigh University, Bethlehem, Pennsylvania, United States of America; 33 Biology Department, The College of St. Scholastica, Duluth, Minnesota, United States of America; Centro Nacional de Biotecnologia - CSIC, SPAIN

## Abstract

Mycobacteriophages – viruses of mycobacterial hosts – are genetically diverse but morphologically are all classified in the Caudovirales with double-stranded DNA and tails. We describe here a group of five closely related mycobacteriophages – Corndog, Catdawg, Dylan, Firecracker, and YungJamal – designated as Cluster O with long flexible tails but with unusual prolate capsids. Proteomic analysis of phage Corndog particles, Catdawg particles, and Corndog-infected cells confirms expression of half of the predicted gene products and indicates a non-canonical mechanism for translation of the Corndog tape measure protein. Bioinformatic analysis identifies 8–9 strongly predicted SigA promoters and all five Cluster O genomes contain more than 30 copies of a 17 bp repeat sequence with dyad symmetry located throughout the genomes. Comparison of the Cluster O phages provides insights into phage genome evolution including the processes of gene flux by horizontal genetic exchange.

## Introduction

The bacteriophage population is vast, dynamic, and old, spanning considerable genetic diversity [1–3]. Phages of phylogenetically distant hosts typically share little nucleotide sequence similarity and few genes encoding proteins with amino acid sequence similarity [4]. Phages also typically encode a high proportion of genes with no sequence similarity to proteins outside of the phages of that particular host, and the global phage population likely harbors the largest reservoir of unexplored sequence information [5]. Phages of a single common host may also show substantial nucleotide sequence variation, although the diversity is expected to be dependent on the diversity of the bacterial population within the environment from which those phages are isolated [6].

Mycobacteriophages—viruses of mycobacterial hosts—display considerable genetic diversity and GC% content [7, 8]. Comparative genomics of over 290 fully sequenced mycobacteriophage genomes shows that they can be divided into groups of closely-related genomes referred to as clusters, several of which can be further divided into subclusters. [7]. There are currently 20 clusters (A-T) and nine singleton phages (those without any close relatives), and ten of the clusters are subdivided into subclusters (phagesdb.org). The diversity of these phages varies among these various groups, with some containing closely related genomes sharing >90% of their genes, whereas others are highly diverse. The genomes are typically mosaic in their architectures, with individual genes or groups of genes present in a multitude of different genomic contexts [9].

Mycobacteriophage Corndog was isolated using *M*. *smegmatis* mc^2^155 as a host and was previously described as a singleton phage with an unusual prolate head [9]. The vast majority of mycobacteriophages have siphoviral morphologies, most of them with isometric heads. The exceptions are Corndog and the phages in Cluster I, although their dimensions differ; the length:width ratio of the capsids is 2.5:1 and 4:1 for Cluster I phages and Corndog respectively [8]. Corndog is also unusual in that the viral genome contains an atypically short (4-base) 3’ single strand extension, and appears to use non-homologous end joining to recircularize the genome upon infection, a process likely facilitated by a phage-encoded Ku protein [10]. Corndog does not infect *M*. *tuberculosis* or *M*. *smegmatis* Jucho, and plates at a greatly reduced efficiency on *M*. *smegmatis* MKD8 relative to *M*. *smegmatis* mc^2^155 [6]. The genome was noted to contain several unusual features including genes coding for methylases and glycosylases within the structural genes, a DNA Polymerase Beta clamp, and an AAA ATPase [9]. Corndog does not encode an integrase and stable lysogens have not been reported [8].

Here we describe four mycobacteriophages—Catdawg, Dylan, Firecracker, and YungJamal—with strong nucleotide sequence similarity to phage Corndog such that all five genomes constitute Cluster O. These genomes are sufficiently similar that dividing the cluster into subclusters is not warranted, and all five exhibit the prolate capsid morphology described for Corndog [9]. Genome comparisons reveal several notable features including putative transcriptional promoters and an unusual 17 bp repeated motif present more than 30 times in each genome. Proteomic analysis of purified Corndog virions and Corndog infected cells identifies about half of the predicted gene products including many small non-structural proteins of unknown function and one previously unannotated gene. Additional proteomic analysis of an unpurified lysate of Catdawg virions identifies a similar proportion of the predicted gene products.

## Results

### Five mycobacteriophages constitute Cluster O

**Fig 1:**
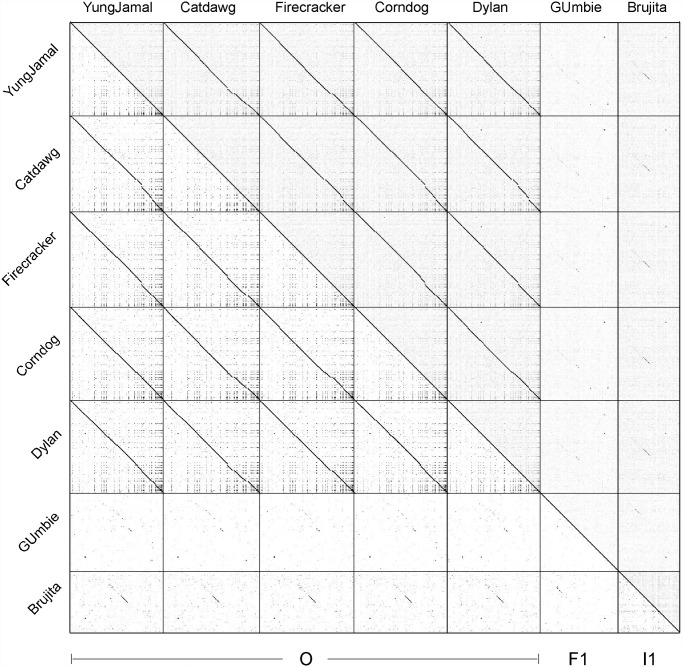
Dotplot comparison of Cluster O mycobacteriophages. The five Cluster O phages along with GUmbie (Subcluster F1) and Brujita (Subcluster I1) were compared using Gepard [13] and the dotplots displayed at two different levels of sensitivity and contrast in the upper right and lower left triangles.

Mycobacteriophage Corndog was isolated in 2001 [9] and until 2012 was designated as a singleton phage without any close relatives [11]. Since 2012, four phages—Catdawg, Dylan, Firecracker, and YungJamal—have been found that are related to Corndog and constitute Cluster O (Table 1, Fig. 1). They were isolated in the Science Education Alliance Phage Hunters Advancing Genomics and Evolutionary Science (SEA-PHAGES) program [12], the Mycobacterial Genetics Course held at the University of KwaZulu Natal (UKZN MGC) and the Phage Hunters Integrating Research & Education (PHIRE) Program at the University of Pittsburgh. The five Cluster O phages have similar genome lengths (69.8–72.1 kbp) and all contain unusually short (4-nucleotide) 3’ single-stranded terminal extensions (Table 1). They have 122–128 predicted protein-coding genes and do not contain tRNA or tmRNA genes (Table 1). The five genomes are closely related at the nucleotide level (Fig. 1) and share high levels of average nucleotide identity (Table 2) that do not warrant division into subclusters. The Cluster O phages are not closely related to other mycobacteriophages although there is nucleotide sequence similarity to Subcluster I1 phages such as Brujita and to a lesser extent subcluster F1 phages such as GUmbie (Fig. 1). The GC% contents are similar to *M*. *smegmatis* (which is 67.4% GC; Table 1) as are the codon usage profiles (data not shown).

**Table 1:** Cluster O Mycobacteriophages.

Phage Name	Accession #	Genome Length (bp)	GC%	Overhang Sequence	# ORFs	Location
Catdawg	KF017002	72108	65.4	GTGT	128	Radnor, PA USA
Corndog	AY129335	69777	65.4	GTCT	124	Pittsburgh, PA USA
Dylan	KF024730	69815	65.4	GTGT	122	Durban, South Africa
Firecracker	JN698993	71341	65.5	GTGT	127	Santa Cruz, CA USA
YungJamal	KJ829260	70214	65.3	GTCT	124	Pittsburgh, PA USA

**Table 2:** ANI values for cluster O phages.

	Catdawg	Corndog	Dylan	Firecracker	YungJamal
Catdawg	1	0.977	0.978	0.973	0.977
Corndog	0.977	1	0.987	0.987	0.991
Dylan	0.978	0.987	1	0.987	0.982
Firecracker	0.973	0.987	0.987	1	0.985
YungJamal	0.977	0.991	0.982	0.985	1

All five Cluster O phages have similar virion morphologies and are members of the Siphoviridae containing long, flexible non-contractile tails approximately 248±8 nm in length. However, they have unusual prolate heads with a length of 165±2 nm and width of 38±1 nm (length:width ratio of 4:1; Fig. 2).

**Fig 2:**
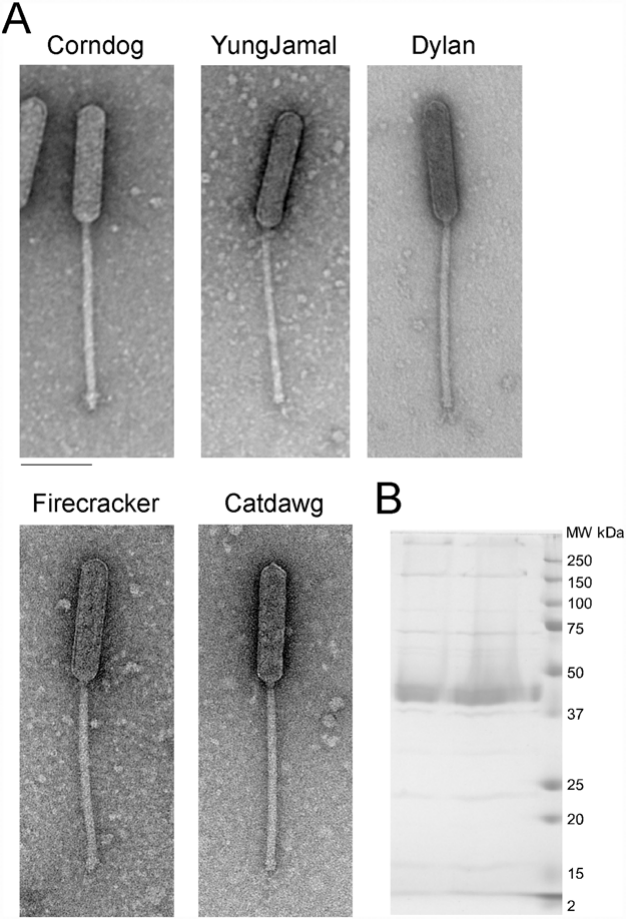
Cluster O mycobacteriophage virion morphologies. **A**. Electron micrographs of Cluster O phages. Scale bar corresponds to 100 nm. **B**. SDS-PAGE analysis of Corndog virions.

### Cluster O Genome Organizations

**Fig 3:**
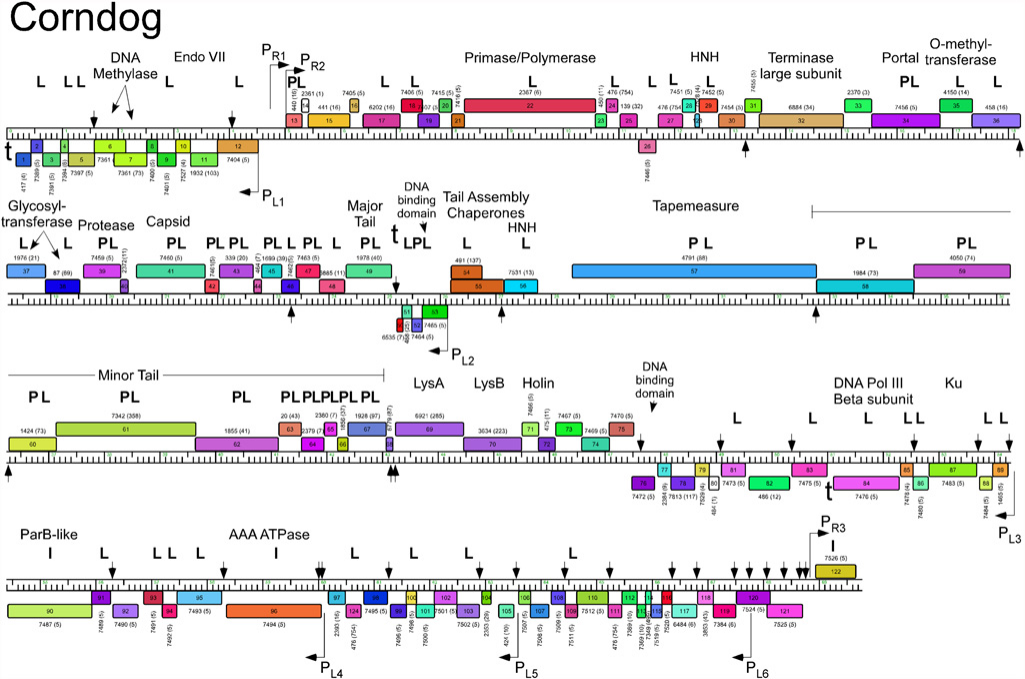
Genome map of Mycobacteriophage Corndog. The genome of phage Corndog is represented as a scale bar (major intervals: 1 kbp) with predicted genes shown as boxes either above (rightwards transcribed) or below (leftwards transcribed). Gene number is shown within each box and the phamily designation is shown either above or below with the number of phamily members shown in parentheses. Putative gene functions are indicated. The positions of putative SigA-like promoters (P_L1_—P_L6_ and P_R1_—P_R3_) are shown as large arrows and terminators (t) are indicated. Small vertical arrows show the locations of the palindromic repeat 5′-TGTTCGGNNNCCGAACA. Gene products identified by mass spectrometry (with at least two high confidence peptides per product) in twice CsCl banded particles (**P**) or from a once-banded lysate (L) are indicated, as well as three additional proteins identified in infected cells (I) not identified in the other samples. Proteins gp11, gp33, gp77, and gp102 had multiple high quality spectra (2, 2, 2, and 4 respectively) of a single peptide each.

**Fig 4:**
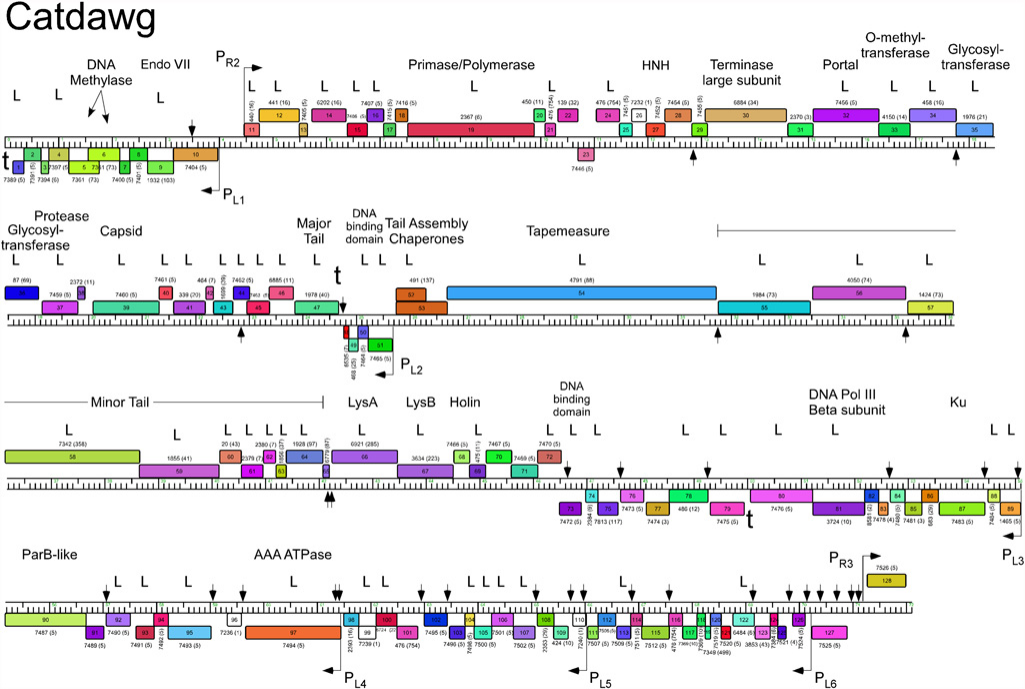
Genome map of Mycobacteriophage Catdawg. The genome of phage Catdawg is represented as a scale bar (major intervals: 1 kbp) with predicted genes shown as boxes either above (rightwards transcribed) or below (leftwards transcribed). Gene number is shown within each box and the phamily designation is shown either above or below with the number of phamily members shown in parentheses. Putative gene functions are indicated. The positions of putative SigA-like promoters (P_L1_—P_L6_ and P_R1_—P_R3_) are shown as large arrows. Small vertical arrows show the locations of the palindromic repeat 5′-TGTTCGGNNNCCGAACA. Catdawg proteins identified in a phage lysate using LC-MS/MS with at least two high confidence peptides per product are indicated (L).

**Fig 5:**
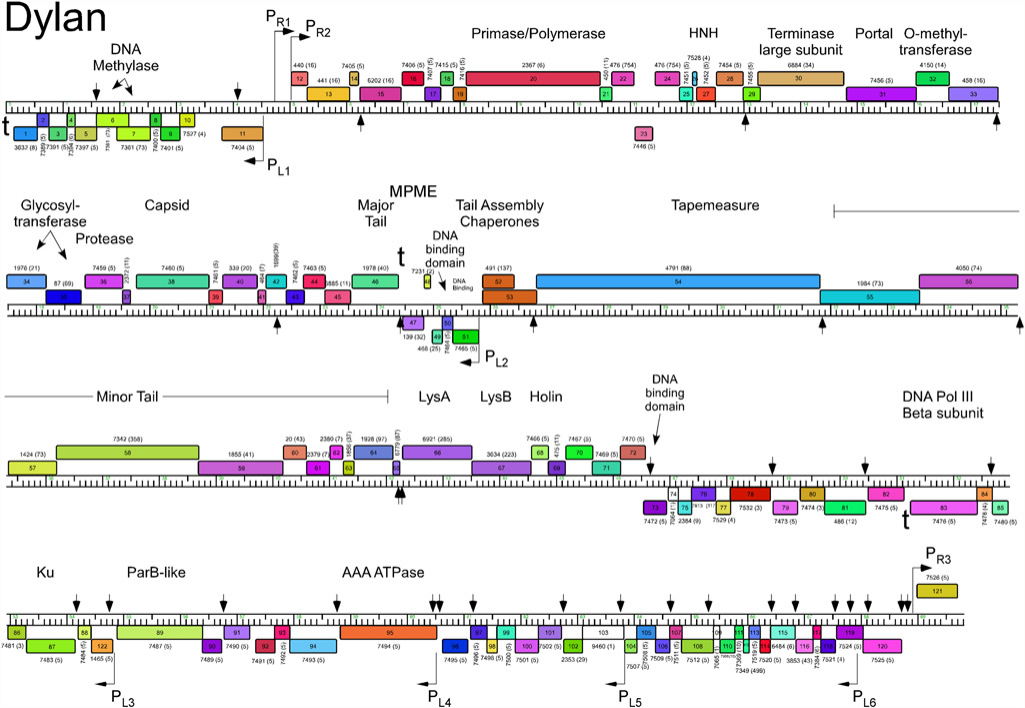
Genome map of Mycobacteriophage Dylan. The genome of phage Dylan is represented as a scale bar (major intervals: 1 kbp) with predicted genes shown as boxes either above (rightwards transcribed) or below (leftwards transcribed). Gene number is shown within each box and the phamily designation is shown either above or below with the number of phamily members shown in parentheses. Putative gene functions are indicated. The positions of putative SigA-like promoters (P_L1_—P_L6_ and P_R1_—P_R3_) are shown as large arrows. Small vertical arrows show the locations of the palindromic repeat 5′-TGTTCGGNNNCCGAACA.

**Fig 6:**
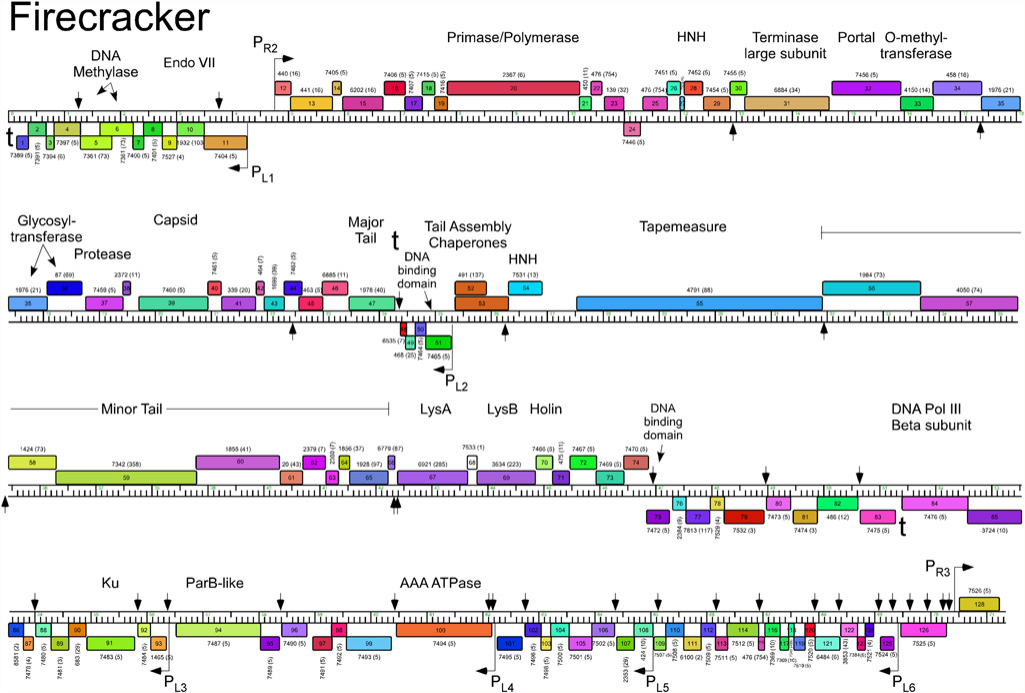
Genome map of Mycobacteriophage Firecracker. The genome of phage Firecracker is represented as a scale bar (major intervals: 1 kbp) with predicted genes shown as boxes either above (rightwards transcribed) or below (leftwards transcribed). Gene number is shown within each box and the phamily designation is shown either above or below with the number of phamily members shown in parentheses. Putative gene functions are indicated. The positions of putative SigA-like promoters (P_L1_—P_L6_ and P_R1_—P_R3_) are shown as large arrows. Small vertical arrows show the locations of the palindromic repeat 5′-TGTTCGGNNNCCGAACA.

**Fig 7:**
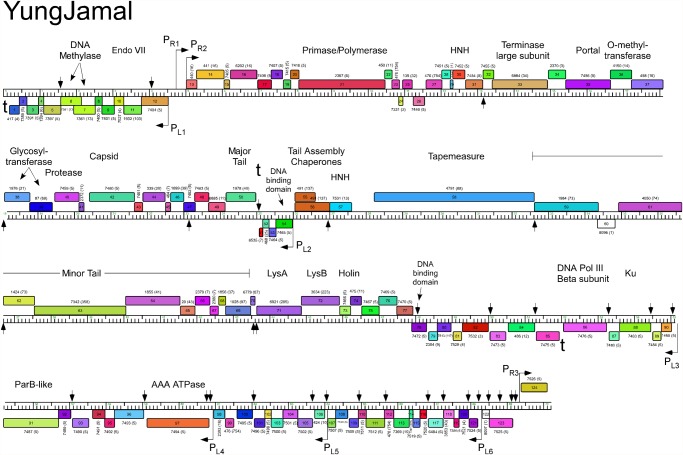
Genome map of Mycobacteriophage YungJamal. The genome of phage YungJamal is represented as a scale bar (major intervals: 1 kbp) with predicted genes shown as boxes either above (rightwards transcribed) or below (leftwards transcribed). Gene number is shown within each box and the phamily designation is shown either above or below with the number of phamily members shown in parentheses. Putative gene functions are indicated. The positions of putative SigA-like promoters (P_L1_—P_L6_ and P_R1_—P_R3_) are shown as large arrows. Small vertical arrows show the locations of the palindromic repeat 5′-TGTTCGGNNNCCGAACA.

The five Cluster O genomes share similar organizations but differ with a variety of small insertions and deletions corresponding to one or a small number of genes (S1 Fig.; Figs. 3–7). The genomes contain three blocks of genes that likely correspond to transcriptional units. The first is a group of 10–12 leftwards-transcribed genes of mostly unknown functions at the left end of the genomes. The second is a large group of rightwards-transcribed genes (e.g. Corndog *11*–*72*) containing the virion structure and assembly genes as well as the lysis cassette, although this is interrupted by up to four instances of a small number of small leftwards-transcribed genes. A third set of ~50 genes (e.g. Corndog *75*–*124*) is transcribed leftwards, and a single gene at the extreme right end of the genomes is transcribed rightwards (Figs. 3–7).

Database comparison and HHPred searches reveal putative functions for fewer than 20% of the genes, although additional virion structure and assembly proteins are predicted based on synteny (Figs. 3–7). Unusually, the large terminase subunit gene is displaced ~14 kbp from the left cohesive end and an O-methyltransferase gene, two glycosyltransferase genes and a putative N-acetylglucosaminyltransferase gene are located between the portal and the capsid maturation protease genes. Of the small leftwards-transcribed genes within the virion structural operon, only one—a putative DNA binding protein (e.g. Corndog *53*)—has a predicted function. Five genes within the long leftwards-transcribed region encode proteins with predicted functions including a DNA binding protein, a beta clamp subunit of DNA Polymerase III, a Ku-like protein, an AAA ATPase, and a ParB-like domain protein.

### Predicted gene expression elements

The prediction of mycobacteriophage promoter locations is complicated because while some are related to mycobacterial SigA promoters [14–16], others appear not to be [17]. However, all five Cluster O phages contain at least eight strongly predicted SigA-like promoters, two rightwards facing (P_R2_—P_R3_) and six facing leftwards (P_L1_—P_L6_); Corndog, Dylan, and YungJamal have an additional rightwards-facing promoter (P_R1_) upstream of P_R2_. P_L1_ and P_R2_ transcribe divergently from the intergenic region located ~5 kbp from the left end and both are predicted to express leaderless mRNAs with the transcription +1 site coinciding with the first base of the first codon of the downstream gene. These intergenic regions are generally much more AT-rich than the rest of the genomes. Promoter P_L2_ that transcribes the leftward facing gene in the structural operon is similarly organized with respect to the start codon of the downstream gene (e.g. Corndog *53*). Four leftwards promoters are situated within the long span of leftwards transcribed genes at the right side of the genomes, suggesting that these constitute at least four separate operons; P_L6_ is within coding regions (e.g. Corndog *120*) but is strongly predicted (5′-TGTCAA—17 bp—TAGAAT).

The Cluster O genomes have three motifs with the potential to form stem-loop RNA structures that play roles in modulating transcription [18]. The first is located at the extreme left ends of the genomes (Corndog coordinates 62–101) such as to terminate leftwards transcription. It contains a 13 bp stem-loop (with a 1 bp bulge) followed by 5′-TTTGT. The second is to the right of the major tail subunit gene (e.g. Corndog *49*; coordinates 25166–25195) and has a 12 bp stem (with a 1 bp bulge), is followed by 5′-TTTCT and likely acts as terminator of rightwards transcription. The third is located between Corndog genes *83* and *84* (Corndog coordinates 51076–51107) and forms a predicted RNA structure with an 18 bp stem and an associated T-rich region that could act as a terminator of leftwards transcription.

### A conserved repeated sequence in Cluster O mycobacteriophages

**Fig 8:**
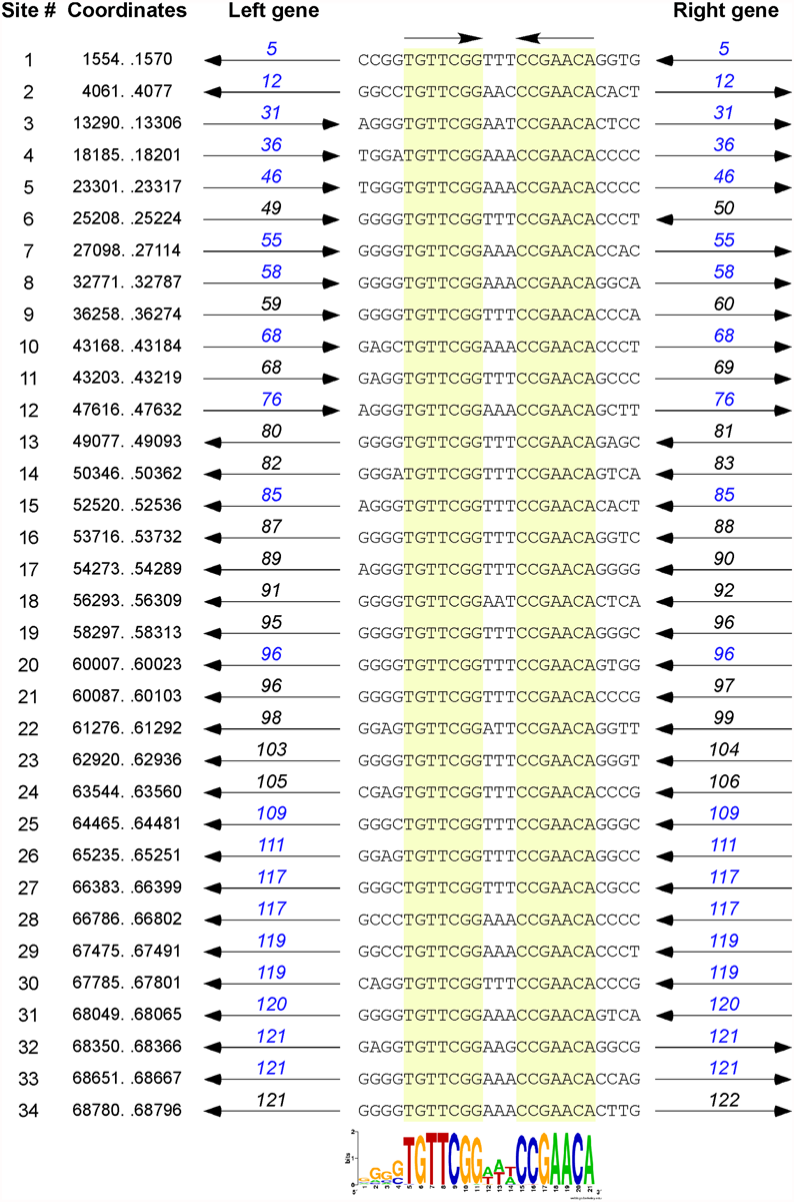
Conserved repeats sequences in the Corndog genome. The Corndog genome contains multiple repeats of a 17 bp sequence composed of two 7 bp inverted motifs separated by three base pairs. The 34 sites are aligned, showing the top strand (and flanking 4 bp) with the 7 bp motifs are highlighted in yellow; the coordinates shown correspond to the 17 bp sequence. The genes flanking the repeat (black) or the genes containing the repeat (blue) and their directions of transcription are shown. Fourteen of the 34 sites (# 6, 9, 11, 13, 14, 16, 17, 18, 19, 21, 22, 23, 24, and 34) are located between open reading frames, ten (#1, 3, 7, 8, 15, 20, 28, 29, 31, and 33) are within open reading frames but close to the 5′ end of the gene (and could be intergenic if the start site is not correctly identified), and ten (#2, 4, 5, 10, 12, 25, 26, 27, 30, and 32) are in the middle or towards the 3’ ends of genes (and the gene is not shown). An additional three sites containing a single base change are not shown. The weblogo at the bottom shows alignment of all 34 sites and related sites identified by MEME [19]; both orientations are compiled due to the inverted repeat such that the flanking 4 bp is shown only on the left. Note that the central three nucleotide spacer is A/T rich, with the most common sequence being AAA or TTT (29 of the 34 sites). There is a slight preference for the orientation of the site to be such that the AAA is on the top strand when the site is transcribed in the rightwards direction. The flanking four nucleotides are G/C rich.

The dot plot genome comparison (Fig. 1) suggests the presence of a small repeated sequence present many times in each of the Cluster O genomes. The conserved 17 bp sequence contains a 7bp inverted repeat separated by 3 bp (5′-TGTTCGGNNNCCGAACA) and is present 34 times in Corndog (Fig. 8) and similarly in the other Cluster O phages. The inverted repeat sequences are invariant among the 34 Corndog sites (there are three additional sites varying at one position), and although there is variation in the central three nucleotides, 5′-TTT (or 5′-AAA) is the most common, present in 29 of the 34 sites (Fig. 8). However, there is little evidence to support meaningful site orientation based on the central trinucleotide asymmetry, at least with regards to the direction of transcription; for example, of the 23 sites within the leftwards operon at the genome right end—Corndog genes *76*–*121*–14 have 5′-TTT and 6 have 5′-AAA on the top strand (Fig. 8).

Most of the sites are in similar positions in all five genomes, although there are informative departures of two types. First, there are several instances where there is apparent loss of a site because of a single base change in one of the repeats. One example is a site in Corndog, Dylan, Firecracker and YungJamal immediately to the left of the methylase genes (e.g. Corndog *6*; Fig. 3), which in Catdawg, has a single base change in the lefthand 7 bp segment. The change is non synonymous for the downstream gene (e.g. Corndog *5*), and the sequence diverges downstream of it. A second example is the loss of a site in Catdawg in the 3’ end of the larger tail chaperone gene (e.g. Catdawg *53*, Fig. 4) because of a change at one position that is synonymous for the reading frame. A second type of departure is where recombination between sites appears to have contributed to insertions or deletions. One example is the presence of a ~550 bp segment between Catdawg genes *95* and *97* that is flanked by two of the repeats. In the other four genomes there is only a single copy of the repeat, and a simple explanation is that Catdawg represents the ancestral state with the other genomes having a deletion resulting from recombination between the two repeats. In a second example, the region immediately downstream of the P_L6_ promoter in Corndog appears to represent the ancestral state with all other genomes having a deletion created by recombination between the two Corndog repeats immediately downstream of P_L6_.

Fourteen of the Corndog repeats are within short intergenic regions and several others are close to the 5′ end of the coding region and the annotated start site choice has yet to be confirmed (see below; Fig. 8). Eleven of the sites are clearly within coding regions (in Corndog genes *12*, *36*, *46*, *55*, *68*, *76*, *108*, *111*, *117*, *120*, and *121*). However, the intergenic sites are not randomly distributed across the genome, and they are predominantly (11 of 14 in Corndog) in the leftwards-transcribed region of Corndog genes *76*–*121* (Fig. 3). The site symmetry suggests these represent binding sites for dimeric regulatory proteins, and we note there are three predicted DNA binding proteins encoded in each of the genomes (e.g. Corndog gp53, gp76, and gp90). However, the possible regulatory consequences are not clear. Although four of the sites are near predicted promoters, most are not, and a transcriptional regulatory function for these repeats seems unlikely. The site is not present in *M*. *smegmatis* mc^2^155 or *M*. *tuberculosis* genomes, or the genomes of other mycobacteriophages; there are two copies in *Mycobacterium sp* 05′1390 [20].

### Identification of Cluster O phage proteins by SDS-PAGE and mass spectrometry

SDS-PAGE analysis of Corndog virion proteins shows a prominent band of 40 kDa and at least six minor proteins (Fig. 2B). Further analysis of CsCl-purified (twice banded) Corndog virions by LC-MS/MS identified twenty-one proteins with high confidence (≥2 peptides/protein Fig. 3, Table 3). All of these are encoded by genes in the interval *34*–*67* with the exception of gp13 (Fig. 3) and include the capsid (gp41) and major tail subunits (gp49), portal (gp34), protease (gp39), putative tail capping and head-tail connector proteins (gp42, gp43, gp45, gp47), tapemeasure protein (gp57) and minor tail proteins (gp58—gp67), as well as gp52 which is of unknown function and transcribed opposite to the other virion genes (Fig. 3). We note that other proteins encoded within this region including the O-methyltransferase (gp35), the glycosyltransferases (gp36, gp37) and the N-acetylglucosaminyltransferase (gp38) were not identified in the virions. LC-MS/MS of Corndog particles purified through a single round of CsCl banding identified all of the same proteins and another 36 Corndog-encoded proteins that are presumably contaminants from lysed cells (Table 3). For an additional four proteins (gp11, gp3, gp77, and gp102) we identified multiple spectra (2, 2, 2, and 4 respectively) but only from a single unique peptide each. We also analyzed extracts of Corndog-infected cells by LC-MS/MS and identified an additional three gene products (gp90, gp96, and gp122) not found in the other samples (Fig. 3, Table 3). The proportion of predicted products identified by LC-MS/MS (48%) is somewhat lower than for similar experiments with mycobacteriophage Patience (75%) [21]. We also analyzed an unpurified lysate of Catdawg by LC-MS/MS using both chymotrypsin and trypsin cleavage (Table 4). A total of 63 proteins were identified (49% of total predicted), with a profile that is similar but not identical to the Corndog proteins.

**Table 3:** Corndog peptides identified by mass-spectrometry.

Coordinates	Product/ Function	Corndog Particles^1^		Infected cells	Total Peptides^2^	Start site Confirmed^3^
		1x CsCl	2x CsCl			
28380–32765	gp 57 tapemeasure	1266	93	100	1459	See text
36294–37142	gp 60 minor tail protein	506	38	32	576	Confirmed
22037–22642	gp 43	444	63	60	567	Confirmed
32803–34521	gp 58 minor tail protein	525	26	13	564	Reassigned
37139–39649	gp 61 minor tail protein	328	61	59	448	Confirmed, acetyl
15549–16778	gp 34 portal	358	35	49	442	Insufficient data
39642–41132	gp 62 minor tail protein	280	35	28	343	Confirmed
25493–25684	gp 52	175	35	49	259	Confirmed, acetyl
19596–20261	gp 39 capsid mat. protease	209	22	23	254	Consistent
24316–25131	gp 49 major tail	174	43	19	236	Confirmed
34518–36254	gp 59 minor tail protein	187	23	5	215	Confirmed, acetyl
41144–41545	gp 63 minor tail protein	144	17	12	173	Confirmed
41960–42184	gp 65	104	3	0	107	Confirmed
41549–41950	gp 64 minor tail protein	89	9	8	106	Confirmed
23417–23842	gp 47	54	5	10	69	Insufficient data
56866–57207	gp 93	52	0	15	67	Confirmed
20547–21779	gp 41 major capsid	54	6	5	65	Confirmed
21779–22027	gp 42	56	5	0	61	Confirmed
42385–43071	gp 67	34	5	1	40	Confirmed
5024–5311	gp 13	31	2	0	33	Confirmed
42197–42385	gp 66	25	3	0	28	Processed?
22796–23155	gp 45	15	5	6	26	Insufficient data
17352–18221	gp 36	22	0	0	22	Confirmed
1111–1581	gp 5	19	0	0	19	Confirmed, acetyl
60504–60770	gp 124	18	0	0	18	Confirmed
26207–26761	gp 54 tail assembly chaperone	18	0	0	18	Insufficient data
11715–12149	gp 27	16	0	0	16	Consistent
12449–12778	gp 29	16	0	0	16	Consistent
6409–7074	gp 17	15	0	0	15	Confirmed, acetyl
23835–24287	gp 48	10	1	4	15	Insufficient data
53972–54247	gp 89	13	0	0	13	Confirmed
57197–57469	gp 94	12	1	0	13	Reassigned
23152–23472	gp 46	12	0	0	12	Insufficient data
18907–19527	gp 38 glycosyltransferase	11	0	0	11	Insufficient data
51112–52290	gp 84 DNA pol Beta subunit	3	0	8	11	Insufficient data
18218–18910	gp 37 glycosyltransferase	10	0	0	10	Insufficient data
25320–25493	gp 51	10	0	0	10	Insufficient data
3790–4524	gp 12	9	0	0	9	Consistent
7098–7472	gp 18	9	0	0	9	Insufficient data
971–1111	gp 4	8	0	1	9	Confirmed
53737–53964	gp 88	8	1	0	9	Insufficient data
11362–11653	gp 26	7	0	0	7	Reassigned
62493–62897	gp 103	6	0	0	6	Insufficient data
64188–64442	gp 109	6	0	0	6	Confirmed
52540–52812	gp 86	5	1	0	6	Confirmed
57466–58266	gp 95	5	0	1	6	Insufficient data
61585–61767	gp 100	5	0	0	5	Insufficient data
49097–49528	gp 81	5	0	0	5	Insufficient data
50364–50996	gp 83	5	0	0	5	Insufficient data
16775–17359	gp 35 O-methyltransferase	4	0	0	4	Confirmed
52318–52539	gp 85	4	0	0	4	Insufficient data
8227–10587	gp 22	3	0	0	3	Insufficient data
10777–10983	gp 24	3	0	0	3	Insufficient data
54424–55932	gp 90 ParB-like	0	0	3	3	Insufficient data
55929–56267	gp 91	3	0	0	3	Insufficient data
58355–60055	gp 96 AAA-ATpase	0	0	3	3	Insufficient data
68942–69664	gp 122	0	0	2	2	Insufficient data
439–651	gp 2	2	0	0	2	Insufficient data
27158–27757	gp 56 HNH endonuclease	2	0	0	2	Confirmed
2707–3042	gp 9	2	0	0	2	Confirmed

^1^Corndog virion particles were purified through one (1x) or two (2x) CsCl equilibrium density gradients.

^2^Table is sorted by total number of peptides assigned by stringent criteria. See text for details and thresholds.

^3^Translation start sites are indicated as confirmed, consistent with the annotation, warranted reassignment of the start site (shown in coordinates), or insufficient data to confirm; acetyl, if more than 50% N-terminal peptides acetylated.

**Table 4:** Identification of Catdawg proteins by mass spectrometry.

Coordinates	Product/Function	Chymotrypsin	Trypsin	Total Peptides^1^	Start site confirmed^2^
23858 to 24673	gp47 major tail	2516	3056	5572	Confirmed
26700 to 31724	gp54 tape measure	1798	1662	3460	Consistent
20089 to 21321	gp39 major capsid	1634	510	2144	Confirmed
31762 to 33480	gp55 minor tail protein	935	963	1898	Confirmed
15091 to 16320	gp32 portal	1039	1134	2173	Consistent
21594 to 22184	gp41	641	1145	1786	Confirmed
33524 to 35260	gp56 minor tail protein	454	763	1217	Confirmed, acetyl
35300 to 36148	gp57 minor tail protein	586	628	1214	Confirmed
36145 to 38655	gp58 minor tail protein	637	399	1036	Confirmed, acetyl
38648 to 40138	gp59 D-ala-D-ala-carboxypeptidase	363	424	787	Confirmed
41391 to 42077	gp64	359	218	577	Confirmed
22338 to 22697	gp43	170	124	294	Confirmed
40150 to 40551	gp60	164	170	334	Confirmed
19138 to 19803	gp37 capsid maturation protease	102	266	368	Consistent
23377 to 23829	gp46	78	139	217	Confirmed, acetyl
40555 to 40956	gp61	124	180	304	Confirmed
22959 to 23384	gp45	113	149	262	Insufficient data
42246 to 43466	gp66 lysA	44	198	242	Confirmed
41203 to 41391	gp63	20	38	58	Insufficient data
40966 to 41190	gp62	3	66	69	Confirmed
6395 to 6769	gp15	3	48	51	Insufficient data
4747 to 5496	gp12		42	42	Insufficient data
25226 to 25035	gp50	8	39	47	Insufficient data
5733 to 6371	gp14	6	28	34	Confirmed, acetyl
16894 to 17763	gp34 glycosyltransferase		34	34	Insufficient data
4467 to 4754	gp11	3	18	21	Confirmed
52190 to 51225	gp81	2	29	31	Consistent
358 to 152	gp1	9	8	17	Confirmed
1198 to 821	gp4	2	28	30	Insufficient data
7521 to 9884	gp19 DNA primase/polymerase		26	26	Insufficient data
46085 to 46525	gp72		18	18	Insufficient data
43468 to 44508	gp67 LysB		19	19	Consistent
46901 to 46494	gp73 HTH DNA binding protein		18	18	Confirmed
25678 to 25223	gp51		21	21	Insufficient data
11045 to 11479	gp24		23	23	Consistent
64667 to 64260	gp106	2	8	10	Consistent
65071 to 64667	gp107		10	10	Confirmed
66856 to 66599	gp113		15	15	Confirmed
61447 to 59669	gp97 AAA ATPase		3	3	Insufficient data
54718 to 54491	gp88		8	8	Confirmed
22709 to 23014	gp44		10	10	Insufficient data
16317 to 16901	gp33 O-methyl transferase	5	6	11	Confirmed, acetyl
59020 to 58220	gp95		9	9	Insufficient data
18449 to 19069	gp36	3		5	Insufficient data
55100 to 54726	gp89		5	8	Insufficient data
49941 to 49309	gp79		8	12	Confirmed
44814 to 45113	gp69		12	6	Insufficient data
**69163 to 68768**	gp122		6	2	Reassigned
57514 to 57065	gp92	2		5	Insufficient data
62494 to 62105	gp100		5	4	Insufficient data
17760 to 18452	gp35 glycosyltransferase		4	7	Insufficient data
47213 to 46980	gp74	7	4	Confirmed	
57961 to 57620	gp93	2	2	4	Insufficient data
62098 to 61778	gp99		4	6	Insufficient data
64263 to 63934	gp105		6	5	Consistent
25749 to 26303	gp52 tail assembly chaperone		5	6	Insufficient data
21321 to 21569	gp40		6	3	Insufficient data
3150 to 2665	gp9 Endo VII protein		3	2	Insufficient data
51220 to 50057	gp80 DNA pol III beta subunit		2	3	Insufficient data
10086 to 10280	gp21		3	2	Insufficient data
6762 to 7073	gp16		2	4	Confirmed
49266 to 48541	gp78		4	2	Insufficient data
63941 to 63762	gp104		2	2	Consistent

^1^Table is sorted by total number of peptides assigned by stringent criteria. See text for details and thresholds.

^2^Translation start sites are indicated as confirmed, consistent with the annotation, warranted reassignment of the start site (shown in coordinates), or insufficient data to confirm; acetyl, if more than 50% N-terminal peptides acetylated.

The LC-MS/MS analysis unfortunately provides few clues as to the basis of the prolate capsids of the Cluster O phages. The capsid subunits (Corndog gp41) are predicted to be structurally similar to the isometric HK97 capsid subunit by HHPred [22] analysis, including the N-terminal 102-residue delta domain that is cleaved and lost during capsid maturation [23, 24]. The LC-MS/MS analysis reveals very few Corndog capsid subunit peptides from either purified particles or late-infected cells, perhaps reflecting poor trypsin digestion of the high molecular weight covalently crosslinked protein seen by SDS-PAGE (Fig. 2B), as seen in HK97 [25]. However, two of the six Corndog virion capsid peptide spectra identified correspond to the delta domain suggesting that it may remain during capsid maturation. Poor recovery of capsid peptides could also result from modifications whose masses are not readily predictable—such as complex sugar additions—and escape LC-MS/MS deconvolution. Major capsid subunit peptides were well-represented in the Catdawg sample, but many of these could have come from unassembled procapsids. We note that six Corndog proteins (gp5, gp17, gp52, gp59, gp61) and five Catdawg proteins (gp14, gp33, gp46, gp56 and gp58) have N-terminally acetylated peptides all at a threonine encoded by the second codon. The functional consequences of this—if any—are not known.

In general, the LC-MS/MS analysis provides information about the translational start sites, and for 26 Corndog genes the annotated start site is confirmed (Table 3), and in 4 others the data is consistent with the predicted start but does not discern between the predicted start site and other possible start sites. For three genes (Corndog *26*, *58*, and *94*) the LC-MS/MS data support re-annotation of the start sites (to positions 11,653, 32,803, and 57,469 respectively; Table 3). For one protein, Corndog gp66, 28 peptide spectra were obtained, but all correspond to the C-terminal 34 residues of the predicted 62-residue product suggesting that it may be post-translationally processed (Table 3). For its Catdawg homologue (gp63), 58 spectra were recovered all of which—with one exception that could be derived from an uncleaved precursor—are in the same C-terminal moiety. We also identified peptides for a previously unannotated Corndog gene (*124*) encoded between genes Corndog *97* and *98* (Table 3).

LC-MS/MS data confirms annotated start sites for 26 Catdawg genes and in nine others the data is consistent with the predicted start does but does not discern between the predicted start site and other possible start sites (Table 4). For one gene (Catdawg *122*) the LC-MS/MS data support re-annotation of the start site to position 69163 (Table 4).

**Fig 9:**
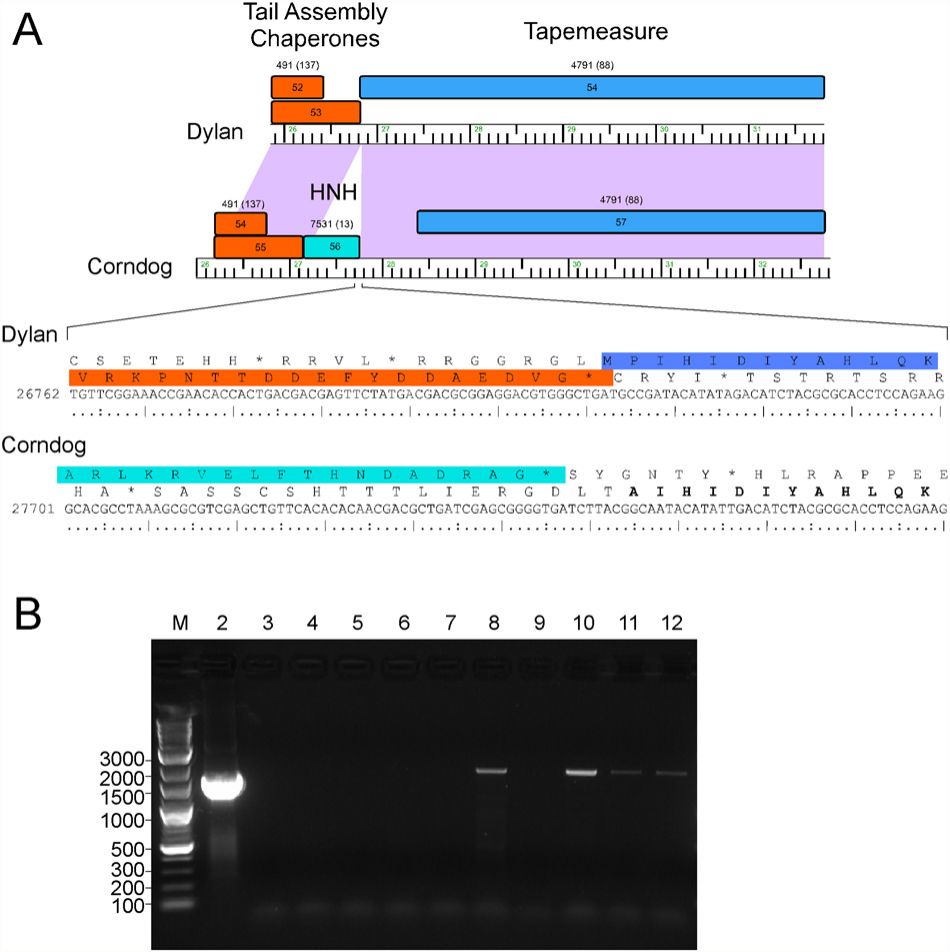
Unusual translation initiation of the Corndog tape measure protein gene. **A**. Two organizations of the tape measure genes are present in the Cluster O phages. In Dylan and Catdawg the *tmp* gene is predicted to start translation immediately downstream of the tail assembly chaperone genes that are translated via a programmed translational frameshift. In contrast, Corndog, Firecracker, and YungJamal have a non-coding gap prior to the *tmp* start site. However, LC-MS/MS identified Corndog peptides corresponding to this gap and the sequence of the most N-terminal peptides are shown in bold type. Translation presumably either initiates at the ACG threonine codon or starts further upstream and involves a ribosome bypass event. **B**. RT-PCR of Corndog transcripts. PCR products were generated using a Corndog lysate (lane 2) or RNA isolated from uninfected cells (lanes 3 and 4), or at different times after infected by Corndog: 30 min (lanes 5 and 6), 2.5 h (lanes 7 and 8), 3.5 h (lanes 9 and 10), and 4.5 h (lanes 11 and 12. Lanes 3, 5, 7, 9, and 11 are controls lacking reverse transcriptase. A DNA ladder marker (M) is shown with sizes in base pairs. Genomic DNA and unspliced RNAs generate an expected product of ~1.7 kbp. No smaller spliced products are observed.

Alignment of the Cluster O genome maps (S1 Fig., Figs. 3–7) shows an evident disparity in the annotation of the tape measure protein (*tmp*) genes. In Catdawg and Dylan the predicted translational start site overlaps the termination codon of the upstream tail assembly chaperone gene, and the LC-MS/MS data are consistent with the annotated Catdawg tmp start site (Table 4). However, in Corndog, Firecracker, and YungJamal, an HNH gene is inserted between the tail assembly chaperone and *tmp*, resulting in *tmp* being annotated to begin at the first available start codon ~ 600 bp downstream, leaving a non-coding gap (Fig. 9A). However, LC-MS/MS of Corndog proteins identified many peptide spectra corresponding to the upstream region of the *tmp* ORF indicating that translation begins upstream. The most N-terminal peptides have the sequence N-AIHIDIYAHLQK and are not generated by tryptic digestion. There are no canonical translation start sites between these peptides and the most upstream termination codon (Fig. 9A), and the threonine codon immediately upstream of this peptide (5′-ACG) is in the corresponding position to the tmp 5′-ATG start codon in Dylan and Catdawg (Fig. 9A). We have been unable to identify any RNA-level splicing event that would suggest that the HNH gene is part of an intron (Fig. 9B) and the most likely possibilities are that either the 5′-ACG codon is used for translation initiation or that translation begins upstream and tmp translation involves a ribosome bypassing event [26]. We are not aware of any other mycobacterial genes initiating translation with ACG and attempts to sequence the tmp N-terminus by Edman degradation have failed, presumably due to modification; the five N-terminal residues from another protein (gp43) from the same gel were readily determined.

### Mobile Elements in Cluster O phages

We noted previously that Corndog contains a truncated version of a Mycobacteriophage Mobile Element (MPME) (encoding Corndog gp25) found in phage genomes within an assortment of clusters (27). MPMEs are small (~440 bp) and include a 123-residue ORF, and two types (MPME1 and MPME2) have been described [27]. Phage YungJamal shares the same sequence as Corndog, which includes the left inverted repeat (IR-L) and 363 bp of MPME1, whereas Catdawg and Firecracker contain a similar segment of the MPME element but have different flanking sequences reflecting deletions of the Corndog sequence. Dylan does not contain an MPME fragment at this site but also does not simply correspond to a pre-integration site either, as there is a 20 bp separation between the Corndog/Dylan homology and IR-L rather than the typical 6 bp [27].

**Fig 10:**
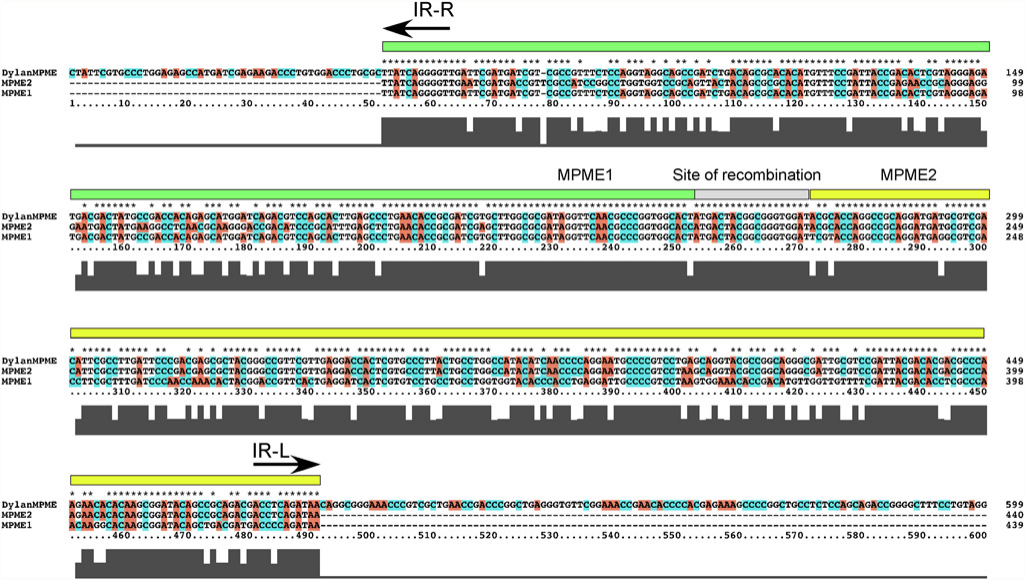
Dylan MPME element. Phage Dylan contains a Mycobacteriophage Mobile Element (MPME) inserted between genes *46* and *48*. The Dylan MPME contains an open reading frame (*47*) that is transcribed leftwards, such that the MPME left inverted repeat (IR-R) is 48-proximal. Alignment of the Dylan MMPE sequence with MPME1 and MPMP2 [27] shows that one half (green box) is identical to MPME1 and the other half (yellow box) is identical to MPME2. The Dylan MPME is thus a hybrid of MPME1 and MPME2, presumably generated by homologous recombination with the intervening sequence (grey box).

Interestingly, Dylan contains a complete MPME element inserted to the right of the major tail subunit gene, and oriented in the opposite direction (i.e. IR-R proximal to the major tail subunit gene; Fig. 5). This MPME element is an apparent hybrid between MPME1 and MPME2 sequences presumably generated by recombination such that the 5′ half corresponds to MPME1 and the 3’ half to MPME2 (Fig. 10). The IR-L of this MPME element (at coordinate 24957) is separated by 6 bp from sequence identity in Corndog (coordinate 25300) and the other phages, indicating this to be the site of the insertion. At the opposite end, there are 14 bp between IR-R and the shared sequences suggesting either differences in the pre-integration site or rearrangements associated with the insertion.

All five Cluster O genomes contain a homing endonuclease-like gene (HNH) gene upstream from the terminase (e.g. Corndog 29) implicated in DNA packaging [28], and two additional HNHs are present in subsets of the genomes. One of these corresponds to the insertion upstream of the tape measure protein gene as discussed above; the other is present in three of the genomes (Corndog, Catdawg, YungJamal) located downstream of the large terminase subunit gene (e.g. Corndog *33*). Dylan and Firecracker lack this HNH gene and comparisons suggest a simple insertion 1–3 bp downstream of the terminase stop codon.

### Other features of Cluster O genomes

There are several other notable features of the Cluster O genomes. First, at the left ends of the genomes there are two adjacent leftwards-transcribed genes coding for domains of cytosine methyltransferases (Corndog genes *6* and *7* and their relatives). Corndog gp7 has a strong HHPred match to the N-terminal part of *Hae*III methylase as well as BLASTP matches to other methylases (including those not encoded by mycobacteriophages) extending across the entire protein span of gp7 (~195 residues) to within a few residues of the gp7 C-terminus. The 53 C-terminal residues of Corndog gp6 (and relatives) are predicted strongly by HHPred to correspond to the three C-terminal alpha helices of *Hae*III methylase. However, the start site of gene *6* is ambiguous, and not only is it the strongest ribosome binding site associated with a start site located within the upstream (e.g. Corndog gene *7*) open reading frame (Figs. 3–7), but there is also coding potential in the gene *6* frame in the overlap region, notwithstanding convincing conservation of the C-terminus of gp7 with numerous methylases. It is thus unclear whether two products are made that assemble to form a methylase active site—and if so where gp6 initiates from—or if a single product is expressed from a translational frameshift, a ribosome hop, or a spliced intron. However, RT-PCR analysis shows no evidence of splicing in this region (data not shown), and products of these genes were not identified by mass spectrometry. We note that similar arrangements of methylase gene segments are seen in other mycobacteriophages, and in phages of other hosts [29].

Secondly, the Cluster O phages encode several proteins with predicted transmembrane domains. Most contain only a single predicted membrane spanning domain and may not be membrane associated. However, downstream of the lysis cassette are two genes (e.g. Corndog *73* and *74*) each encoding products with four predicted transmembrane domains that are strongly predicted to be membrane associated. Neither have relatives in other mycobacteriophages, and their roles are unclear although they could also play a role in lysis.

**Fig 11:**
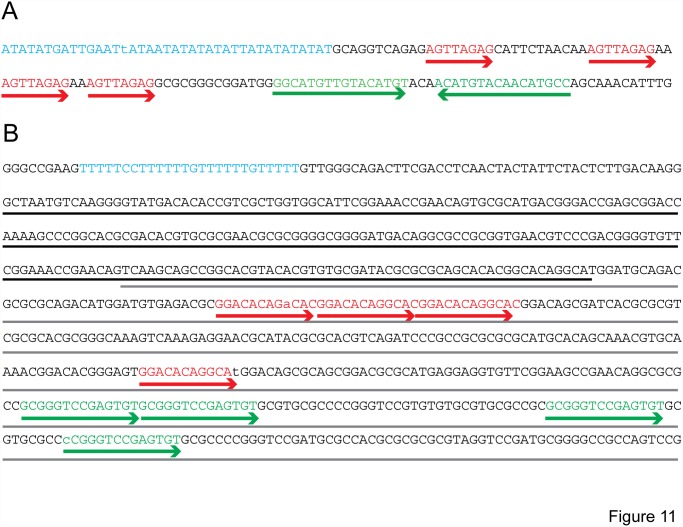
Sequence features of Cluster O genomes. **A**. The AT rich element between Corndog genes *12* and *13* is highlighted in cyan, and two sets of flanking sequence repeats are shown in red and green. A similar arrangement of these sequences is observed in the other Cluster O phages. Residues in these sequence elements that differ across the phages (in the case of the AT rich element), or from the repeat consensus sequences are shown in lower case. **B**. A portion of Corndog genes *120* (underlined in black) and *121* (underlined in gray). The conserved T_5_CCT_6_GT_6_GT_5_ sequence is shown in cyan and flanking sequence repeats are shown in green and red. Residues in these sequence elements that differ across the phages (in the case of the T rich element), or from the repeat consensus sequences are shown in lower case.

The Cluster O genomes contain two AT-rich sequences, which are unusual among the GC rich mycobacteriophage genomes. The first, in the gap between the divergently transcribed operons on the lefthand side of the genome (i.e. Corndog genes *12* and *13*) is a 39 nucleotide sequence consisting of 37 A or T residues that varies at only a single residue across the 5 Cluster O genomes. The second AT-rich sequence occurs at the far right hand side of the Cluster O genomes. In Corndog, this sequence lies within gene *120* whose central part is AT-rich and includes the sequence 5′-T_5_CCT_6_GT_6_GT_5_. Corndog *120* is poorly conserved among Cluster O genomes and we did not observe any peptides that could be encoded by this sequence in our MS data, raising the question of its assignment, but this AT-rich sequence is identical in all five phages. It is located 35 bp downstream of the putative P_L6_ promoter and could play a regulatory role. Interestingly, a complex set of sequence repeats occurs to the right of each of these AT-rich elements (Fig. 11), and it is plausible that one or the other of these represents the phage origin of replication.

### Insights into phage genome evolution

Several regions of the Cluster O genomes differ in gene content as a consequence of deletions or insertions, typically by one or a small group of genes. These gene content differences occur in a variety of genomic contexts and apparently reflect relative recent horizontal exchange events rather than whole genome ancestries.

**Fig 12:**
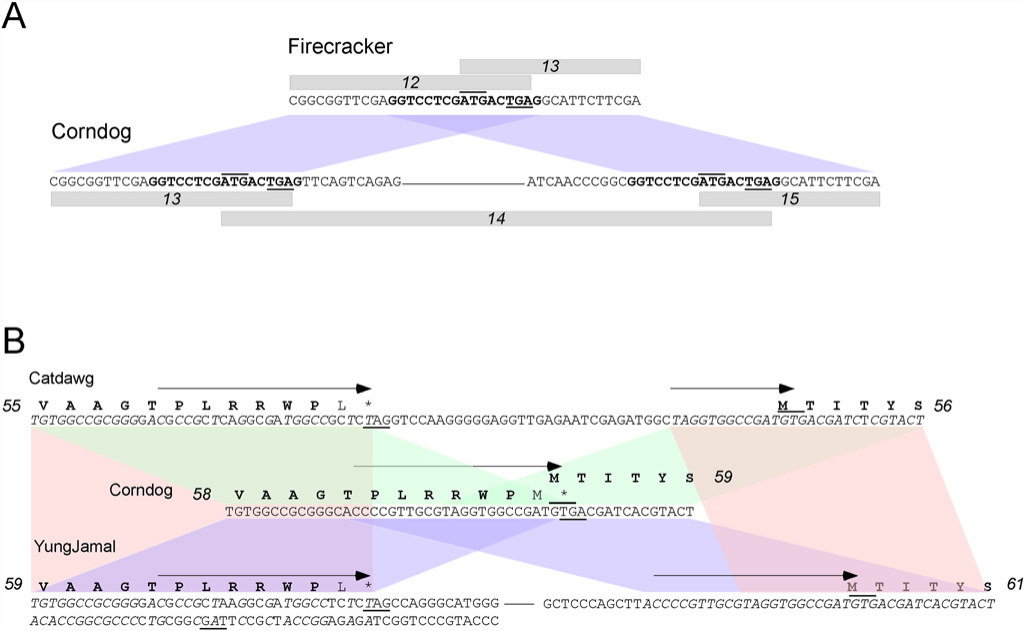
Insights into genome evolution. **A**. Insertion of Corndog gene *14*. Cluster O genome comparisons show that Corndog gene *14* is missing from the other four related genomes. A 17 bp direct repeat (bold type) flanks Corndog gene *14* but is present only once at the junction of Firecracker gene *12* and *13* and their homologues in Dylan, Catdawg and YungJamal. Termination codons are underlined and translation start codons are overlined. Regions of nucleotide similarity are indicated by colored trapezoids. **B**. Insertion of gene *60* in YungJamal. YungJamal gene *60* encodes a protein of unknown function and is absent in all four other Cluster O genomes. YungJamal *60* is transcribed leftwards and is flanked by imperfectly conserved 24 bp inverted repeats (shown by arrows), but in which only 14 bp are conserved. However, Corndog (as well as Dylan and Firecracker) contains just a single copy of this repeat at the junction of genes Corndog *58* and *59*. Unusually the rightmost copy of the repeat in YungJamal (at the beginning of gene *61*) is identical to the Corndog sequence, whereas the leftmost repeat (at the end of gene *59*) is a degenerate copy in which most of the base changes are synonymous, except for the C-terminal residue. Termination codons are underlined and translation start codons are overlined; the sequences of both strands for the left component of YungJamal are indicated to show the termination codon (underlined) of YungJamal *60*. Catdawg lacks a homologue of YungJamal *60* but carries a small insertion relative to Corndog, Dylan, and Firecracker, and has part of the rightmost YungJamal repeat. Catdawg and YungJamal sequences shared with Corndog are shown in italic type. Sequences of nucleotide similarity are indicated by the colored trapezoids (Catdawg and Corndog, green; Corndog and YungJamal, purple; Catdawg and YungJamal, red).

There are two examples of a gene present in one genome but absent from the other four genomes. Corndog gene *14* is small (126 bp) but HHPred analysis confidently predicts that gp14 folds similarly to the mycobacteriophage Pukovnik Xis protein [30] and is likely to be a DNA binding protein. Genome comparisons show that Corndog *14* is flanked by a 17 bp direct repeat present only once in the other genomes (Fig. 12A). Either Corndog represents the ancestral state from which gene *14* has been deleted by homologous recombination between the repeats, or Corndog has acquired *14* by recombination with a partner DNA carrying a sequence similar to the repeat.

A more complex relationship is seen with YungJamal gene *60*, which is absent from the other four genomes. YungJamal *60* is transcribed in the leftwards direction, opposite to the tail genes that flank it and is of unknown function (Fig. 7). The gene is flanked by imperfect 24 bp direct repeats of which just 14 bp are conserved, and Corndog, Firecracker and Dylan each contain only a single copy of the repeat that is identical to the rightmost YungJamal copy (Fig. 12B). The base differences between the leftmost copy of the repeat in YungJamal and Corndog are such that the amino acid sequence of the products is maintained, with the exception of the C-terminal most residue (Fig. 12B). Catdawg differs from Corndog, Firecracker and Dylan in that it contains a small insertion including a partial second copy of the repeat. A plausible scenario is that Corndog, Firecracker and Dylan represent the ancestral state (and a canonical virion structural gene organization) into which YungJamal *60* was acquired by recombination, which subsequently underwent deletion to give the Catdawg structure. This then provides an evolutionary context for understanding the Catdawg genome that would not have been possible without the other Cluster O relatives.

Among the various other insertions and deletions, we note that Corndog *10* and its homologues in Firecracker and YungJamal are absent from Catdawg. The deletion in Catdawg reflects a loss of 281 bp relative to the other genomes, and an accompanying insertion of 15 bp of unknown origin. There are no obvious repeated sequences flanking the deletion and the mechanism involved is unclear.

## Discussion

The Cluster O mycobacteriophages are an interesting group of phages with several features not found in other phages of *M*. *smegmatis*. The most obvious of these is their prolate heads with a 4:1 length:width ratio. Prolate-headed phages within the *Caudovirales* are somewhat uncommon, with the best-studied being T4, although the length to width ratio of T4 is relatively small. However, phages with longer heads have been described for other hosts including phages of *Caulobacter* (length:width ratios of 3.5:1–4.5:1) [31] and *Lactobacillus* [32–34] and a model has been described for the structural organizations of icosahedral prolate capsids [35]. It is notable that HHpred predicts a subunit fold that is very similar to that of HK97, which forms an isometric shell [36]. The genomic and proteomic analyses identified no unusual components of the particles, such as proteins that might specifically determine capsid length, as tape measure proteins do with tails. The prolate shape thus might be determined solely by the physical nature of the capsomers [35].

Mass spectrometry reveals an unexpected dearth of Corndog capsid peptides, as capsid monomers are expected to be the most abundant components of purified virions. Thirteen virion proteins had more peptides than the capsid subunit, including most of the minor tail proteins, the portal, and the proposed capsid protease. Although it is plausible that some peptides were not identified because of covalent crosslinking as in HK97, it is possible that the mature capsid subunits are modified such as to obscure the predicted peptide masses. Four genes between the portal and protease genes have plausible modification functions including an O-methyltransferase (Corndog gp35), glycosyltransferase proteins (gp36, gp37), and a putative N-acetylglucosaminyltransferase (gp38). All four were identified by LC/MS-MS in infected cells and could add complex methyl and glycan modifications to the capsid with unpredictable molecular masses.

The Cluster O phages carry an unusual array of 17 bp repeats of unknown function. They are located throughout the genomes but are more densely positioned towards the right genome ends. Many are intergenic, although about one-third of them are within coding regions. They differ from the Start Associated Sequences (SAS) repeats in the Cluster K phages [37] in not being closely linked to translational initiation sites, and are more similar in their distribution to the stoperator sites in the Cluster A phages [16, 38]. However the Cluster A stoperator sites are asymmetric and orientated with the direction of transcription, an important feature of their proposed function in termination of transcription and silencing [16]. Moreover, we have not been able to recover stable lysogens of Corndog or other Cluster O phages, and they do not encode an integrase or a parAB partitioning system (the parB-like domain proteins such as Corndog gp90 are unlikely to be involved with genome stability) and are not obviously temperate, at least in *M*. *smegmatis* mc^2^155. However, the sites clearly have dyad symmetry and are predicted to be bound by dimeric DNA binding proteins. Because the large majority of sites are not associated with predicted promoters, the DNA binding interaction must be involved in a process other than the regulation of transcription initiation. We also note that few, if any, of these short repeats are involved in any of the insertions, deletions or rearrangement observed between the five Cluster O genomes.

Finally, comparative genomics and LC-MS/MS resolve the oddity of an apparent extended non-coding gap in Corndog between the tapemeasure protein gene and the upstream gene, which was similarly predicted in the Firecracker and YungJamal genomes. All three also share the insertion of an HNH gene upstream of this apparent non-coding gap. LC-MS/MS analysis shows that translation does indeed begin upstream, although where translation initiates remains unclear, and we have been unable to determine the N-terminal sequence of the tape measure protein by Edman degradation (data not shown). Because there is no commonly used start codon (ATG, GTG, TTG) upstream of the most N-terminal peptides identified, *tmp* expression must use a non-canonical mechanism. Among the possibilities is the use of an unusual codon for translation initiation—perhaps the ACG codon immediately upstream of the N-terminal peptide—or by initiation of translation somewhere upstream coupled with a translational bypass event. Regardless of which non-canonical mechanism is used, there is no obvious reduction in the expression level of *tmp* in Corndog, and the three phages with this arrangement (Corndog, Firecracker, and YungJamal) grow similarly to Catdawg and Dylan that use an ATG start codon.

In summary, the Cluster O mycobacteriophages represent an interesting group of closely related phages with a variety of interesting genomic features. The identification of a variety of conserved features suggests novel and interesting regulatory features warranting experimental investigation.

## Materials and Methods

### Electron Microscopy

Cluster O phage samples were spotted on 400 mesh carbon coated copper grids, stained with 1% uranyl acetate, and imaged with a Morgagni TEM.

### Bioinformatic analyses

Bioinformatic analyses used DNA Master (http://cobamide2.bio.pitt.edu/), Aragorn [39], Gepard [13], HHpred [22], tRNAscan [40], and Phamerator [41]. The Phamerator database used for genomic comparisons was Mycobacteriophage_292. Phams were built using BLASTP and/or ClustalW, with similarity cut-offs e-values of 10^-50^ and 32.5% similarity or better as described elsewhere [41]. Transmembrane domains were identified using SOSUI [42], TopPred [43] and TMHMM [44]. Predicted SigA-like promoters were identified using promoter prediction in DNAMaster set to search for sigma-70 binding sites. The search parameters were as follows: site and merge methods set to geometric, -35 and-10 weights set to 1.0, and spacing weight set to 0.1. The top scoring promoters were evaluated for transcriptional direction of flanking genes and whether they were within or between predicted coding regions.

### SDS-PAGE

Corndog particles were concentrated and purified by CsCl density gradient ultracentrifugation. The visible phage band was dialyzed against two changes of phage buffer (10 mM Tris pH 7.5, 10 mM MgSO_4_, 20 mM NaCl, 1 mM CaCl_2_); 500 μl of the dialyzed CsCl band was pelleted by centrifugation for 30min at 14000 rpm. The pellet was resuspended in 75 μl of 20 mM DTT, then 2 μl of 0.5 M EDTA and 1 μl of 1 M MgSO_4_ was added. The phage was disrupted by heating to 75°C for 2 mins, and then sonicated on ice six times for 30 seconds to disrupt the DNA. The sample was mixed with 25 μl 4 x SDS sample buffer and heated in a boiling bath for 3 minutes at 95°C. The sample was electrophoresed through a 12% polyacrylamide gel containing SDS, and stained with Coomassie Brilliant Blue in methanol.

### Transcript analysis

A log phase *M*. *smegmatis* mc^2^155 culture was infected with Corndog particles at a multiplicity of infection (moi) of 3, and total RNA collected at various time points post-infection (30 min, 2.5 h, 3.5 h, and 4.5 h) using the Qiagen RNeasy Mini Kit (Qiagen). RNA was treated with DNase I (Invitrogen) and cDNA was generated using random hexamers and Maxima reverse transcriptase (Fermentas). PCR was used with the following primers to check the size of the cDNA product: (5′ GAAGGTGCCTTCAAGACGGCCG 3’) and (5′ GCGACCACATCGCTGATGCTCTG 3′). A Corndog phage lysate was used as a positive control for PCR.

### Mass-spectrometry

LC-MS/MS analysis was performed on Corndog particles purified by either one or two rounds of banding by CsCl equilibrium density centrifugation. For LC-MS/MS analysis of infected cells, 5 mls of exponentially growing *M*. *smegmatis* mc^2^155 (OD_600_ = 0.4) in 7H9 /ADC was concentrated to a 500 μl volume *via* low-speed centrifugation, and infected with Corndog at a multiplicity of infection (moi) of 100. Phage particles were allowed to adsorb for 15 minutes, then 4.5 mls of fresh 7H9 medium was added, and incubated further with shaking for three hours at 37°C; the OD_600_ was monitored throughout to follow cell growth and lysis. At 165 minutes post-adsorption, a 1-millilter aliquot was removed from the culture, the cells were pelleted *via* centrifugation (1 min, 14K rpm in a microfuge), and the supernatant was removed. The cell pellet was frozen at—80°C, and then shipped overnight on wet-ice to the University of California, Davis Proteomics Core (UCDPC) http://proteomics.ucdavis.edu. There, the cells were lysed *via* a MagnaLyser, the insoluble fraction was removed, and the soluble proteins were precipitated, digested with Trypsin, and cleaned-up using a macro spin-column. The peptides were then separated using an Easy-LC II High-Pressure Liquid Chromatography HPLC system and loaded into a Q-exactive orbitrap mass spectrometer with a Proxeon nano-spray source (Thermo) for tandem ms analysis. Detected spectra and fragmentation profiles were matched against a database comprised of a six-frame translation of the Corndog genome, the annotated proteins of *M*. *smegmatis* mc^2^155, and UniProt using X!Tandem. Peptide matches were analyzed using Scaffold4. Settings used a peptide threshold of 95%, and protein FDR of 1%. For proteomic analysis of Catdawg, a 1 ml aliquot of a phage lysate was pelleted at 14K for 2 hours at 4°C, resuspended in 100 μl of 0.1 M phosphate buffer and shipped overnight on dry ice to MSBioworks (http://www.msbioworks.com/) for mass spectrometry analysis of phage proteins.

For N-terminal analysis of proteins, a Catdawg lysate were labeled with 200 mM TMPP in 20% acetonitrile [45]. Approximately 20 μg of labeled proteins were resolved and separated on a 4–12% Bis Tris SDS-PAGE gel in MOPS buffer and the gel lanes excised into 20 equally sized segments. Gel segments were protease digested using either trypsin or chymotrypsin and analyzed by nano LC-MS/MS with a Waters NanoAcquity HPLC system interfaced to a ThermoFisher Orbitrap Velos Pro. Peptides were loaded on a trapping column and eluted over a 75 μm analytical column at 350 nL/min. The mass spectrometer was operated in data-dependent mode, with MS performed in the Orbitrap at 60,000 FWHM resolution and MS/MS performed in the LTQ. The 15 most abundant ions were analyzed. Mascot DAT files were parsed into Scaffold for validation and filtered to create a non-redundant list. Filtering used a minimum protein value of 99% and peptide value of 50% (Prophet scores), and required at least two unique peptides per protein. Protease peptide data were merged for analysis. Peptide data from the two different proteases were merged using Scaffold4 for subsequent data analysis Settings used a peptide threshold of 95%, and protein FDR of 1%.

## Supporting Information

S1 Fig Comparison of Cluster O genome maps. Genome maps of the five Cluster O phages, Corndog, Catdawg, Dylan, Firecracker and YungJamal were generated by Phamerator using the database mycobacteriophage_292 (41). Genes are shown as boxes above (rightwards-transcribed) or below (leftwards-transcribed) the genome with gene names within the boxes. Phamily assignments for genes are shown above the boxes with the number of phamily members in parentheses. Shading between genomes shows pairwise nucleotide sequence similarity and spectrum colored with violet being the most similar, and red being the least similar but above the threshold BLASTN E value of 10^-5^.
(PDF)
