# Supplementary figures and images for: Comparative Genomics of Cluster O Mycobacteriophages
Source: PLoS One. 2015 Mar 5;10(3):e0118725. doi: 10.1371/journal.pone.0118725 (PMC4351075; doi:10.1371/journal.pone.0118725)

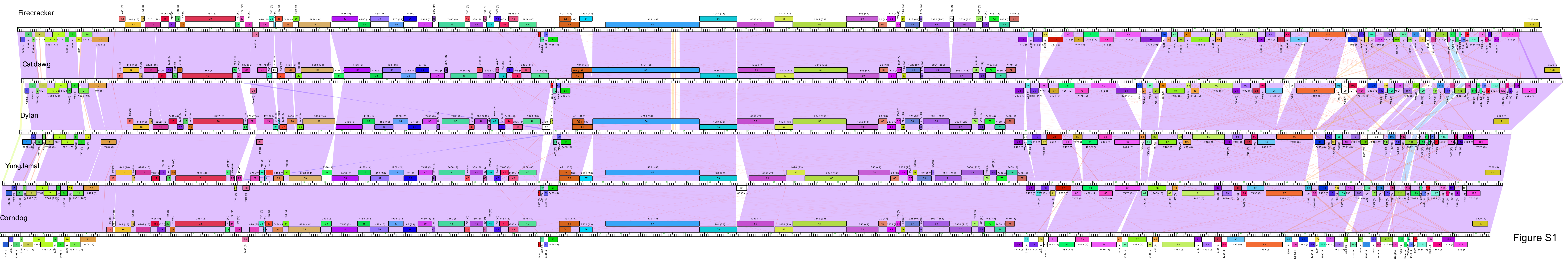

Figure S1

Supplement: S1 Fig — Genome maps of the five Cluster O phages, Corndog, Catdawg, Dylan, Firecracker and YungJamal were generated by Phamerator using the database mycobacteriophage_292 (41). Genes are shown as boxes above (rightwards-transcribed) or below (leftwards-transcribed) the genome with gene names within the boxes. Phamily assignments for genes are shown above the boxes with the number of phamily members in parentheses. Shading between genomes shows pairwise nucleotide sequence similarity and spectrum colored with violet being the most similar, and red being the least similar but above the threshold BLASTN E value of 10-5. (PDF) [file pone.0118725.s001.pdf]
